# Hospital and Institutional News

**Published:** 1919-12-06

**Authors:** 


### HOSPITAL AND INSTITUTIONAL NEWS.

### DR. CECIL LYSTER.

AT the recent meeting of the Court of Governors of Middlesex Hospital a moving reference was made to the resignation due to illness of Dr. Lyster, whose work as an *x*-ray investigator is well known to the medical world. For a number of years Dr. Lyster has been head of the electro-therapeutic department of that hospital, and those who knew him are aware that the apparent deformities of his hands are due to operations following damage by the rays. He was indeed one of the pioneers of research in adapting electrical phenomena to therapeutic uses, and by exposure to the rays when knowledge of their power was slight fell a victim to the distressing lesions they can produce. Though suffering and compelled frequently to seek the surgeon's aid, he declined to be set aside from his investigations and his purpose, and continued his good work until the present time. As the Earl of Athlone remarked, such a record of service to others, a life spent in the advancement of science, with no regard for personal safety, commands our admiration, wonder and respect. We understand that, as we go to press, Dr. Lyster's condition is critical, and we would express our most earnest hope for his recovery.

### REPLENISHED EXCHEQUERS AT THE VOLUNTARY HOSPITALS.

WEEK by week since we began our crusade to lay the ignorant folly of the cry of "Put the Hospitals on the Rates," encouraging evidence of the generous interest taken in our voluntary hospitals has been forthcoming in increasing volume up and down the country. This week it is announced that an anonymous donor has presented £20,000 to the Manchester Royal Infirmary. Will not Manchester adopt Liverpool's example and under the auspices of the Lord Mayor raise a sufficient sum to provide all the voluntary hospitals in Manchester with adequate funds to carry them financially over the three years 1920-22 inclusive? Another instance of the activity of the smaller communities is Gosport, with a population including its suburbs of about 40,000. The local war memorial is a general hospital of forty beds, with an out-patient, *x*-ray, and dental departments up to date. The estimated cost is £25,000, but £50,000 was asked for and £40,000 was reached with gratifying rapidity. Of this, £20,000 was subscribed, £15,000 was added by the Red Cross and St. John's Ambulance Committee, and a site worth £5,000 was presented. It is an interesting feature of this War Memorial that the question of a wooden erection, or one built of a material involving much less cost than the usual brick or stone buildings, is under consideration. We have long given our attention to the subject of wooden buildings for hospital purposes, and have a number of interesting and successful ventures in this direction. The Metropolis of the Empire is waking up in regard to hospital matters, and the developments and new building schemes now in preparation are surprising from their number and variety.

### A WONDERFUL ACHIEVEMENT AND ITS LESSONS

THE account we gave in last week's THE HOSPITAL (page 189) of the remarkable skill and energy developed at Guy's Hospital under the administration of Sir Cooper Perry, with the whole-hearted devotion of the President, Mr. Cosmo Bonsor, has caused universal admiration, and a desire to go and do likewise. Next week we hope to lend our co-operation and help to each hospital committee, and to each hospital worker who may be ambitious to make his or her individual war effort of thanksgiving one to bring aid and strength to the hospital or hospitals in the city, town, or county where they habitually abide and work. Any who have difficulties, all who are anxious to become members of the Crusade of Willing Workers, especially sailors and soldiers who have seen, realised, and possibly benefited by our hospitals during the Greatest War of the World, are invited to place themselves in communication with the Editor, who will gladly put his experience and knowledge at their disposal, and do his utmost to make their personal service in the cause of the hospitals and the sick of supreme interest and uplifting. The Mayors, Chairmen of County Councils and other Leaders of Public Opinion, locally, throughout the United Kingdom have before them in 1920 a splendid opportunity to make their year of office memorable for all time, by putting themselves at the head of a movement, as the Lord Mayor of Liverpool has done, to make the voluntary hospitals of the city, town, or county where these gentlemen have jurisdiction safe for the sick and wounded residents of all classes.

### LADY ASTOR AND CHILDREN'S HOSPITAL CITY.

VISCOUNTESS ASTOR, M.P., is taking the greatest interest in the scheme for the Children's Hospital City, which is being organised by the Hospital for Sick Children, Great Ormond Street, W.C. 1. To-morrow (Thursday, December 4) at 12 noon she will visit the hospital in Great Ormond Street in order to make a distribution of toys to the children in the wards, when it is hoped that she will speak on the scheme of the Hospital City in its relation to the health of the nation.

### THE FEEBLE-MINDED.

IT is to be hoped that the Ministry of Health will tackle the problem of the feeble-minded with a will and without delay. It is one of the most urgent and pressing problems of the day—both from the economic and moral point of view. As is pointed out in a letter to the *Times* of Friday last, a letter with many weighty signatories, now is the time when trouble will surely arise unless the situation is faced. During the war many feeble-minded could find work. It is obvious that they have no chance in present-day competition, and the inevitable result will be not only misery and unemployment, but moral degradation. Naturally, as this letter reminds us, the feeble-minded are much more easily "swayed by vicious influences" and by the deteriorating effect of overcrowding and bad housing than normal persons. We speak here of those who are feeble-minded only and who are not certified under the Mental Deficiency Act of 1913, but who are so far defective as to need great care and supervision in their own interests and in the interests of the public in whose midst they live. This supervision should be placed upon a sound financial footing, and, while no deprecation of the excellent voluntary work which has been done is intended, it is felt that there should be official responsibility for the work. Very great improvements will have to be made in the administration of the Act of 1913, the responsibility of which seems to have been thrown, in London at any rate, in some cases, from the Public Health authority to the Poor-law authority, and it is hoped that when the whole question is investigated by the Ministry of Health, the feeble-minded will receive their full share of attention. Until this is done there is very little hope of eradicating many of the social evils from which we suffer to-day.

### MEASLES NOTIFICATION WITHDRAWN.

The Ministry of Health has published an Order rescinding the Order for the Notification of Measles, issued in 1915. Notification, it is understood, has not resulted in those efforts to control the disease which were hoped for. It is, however, open to any local authority which desires to continue notification to obtain an Order from the Ministry.

### UNHEALTHY AREAS.

It is estimated that an aggregate of 184,000 persons are living in the slum areas of London. Figures given to the Unhealthy Areas Committee of the Ministry of Health at recent meetings go to prove the high mortality brought about by the insanitary condition of these localities. In the Tabard Street area the death-rate between the years 1904 and 1908 was 36.8, as against an average of 14.9 for the rest of London. The Unhealthy Areas Committee are arranging to make a personal inspection of these slums.

### HOSPITAL FINANCE IN GLASGOW.

The managers of the Western Infirmary of Glasgow were far-seeing when they issued a special appeal in 1916-1917 for funds to be held in reserve to be used for maintenance as required, but the extraordinary drain on all resources has at last exhausted this very useful stand-by. The ordinary income last year amounted to £52,948 and the ordinary expenditure to £80,667 leaving a deficit of £27,719, following on a deficit in the previous year of £117,853. Extraordinary income (from legacies and large donations) amounted to £12,154, but extraordinary expenditure was £3,117. The hours of nurses now work out at fifty-six a week, exclusive of the annual holiday, and a larger staff being necessary an addition to the nurses' home must be provided. In the massage department, opened in January 1918, where the training of pupils qualifies for the I.S.T.M. examinations, 306 applications for training have been received.

### THE COST OF DISEASE.

In the last annual report issued by the Local Government Board before its absorption into the Ministry of Health some figures are given which indicate only too plainly how urgent is the need for reorganisation of all health protection measures. The gross expenditure out of the revenue of local authorities on the treatment of tuberculosis from July 15, 1912, to March 31, 1919, is estimated to have exceeded £5,000,000. Venereal disease is also dealt with in relation to its prevention and treatment, and it is noted that already the schemes of 139 local authorities have been approved covering a total of 35,000,000 of population. In 1917 the number of new patients was 27,000; in 1918, 58,000; while the actual attendances in these years numbered 205,000 and 488,000 respectively. In 1918 there were 31,500 new cases of syphilis diagnosed and treated! The exact expenditure on this branch of the work is not to hand, but, knowing the methods of working in these clinics, the quantity and cost of the drugs necessary, and the attendances already noted, it is easy to realise the enormous amount of money involved. These examples, indeed, form but a tithe of the total schemes and total expenditure which are necessarily involved in the attempt to rescue this country from the grip of disease.

### THE PATHOLOGIST'S STATUS.

With reference to the recent prominence which was brought to the fact that, generally speaking, pathologists and bacteriologists were excluded from membership of the medical committees of hospitals, we note that at the weekly meeting of the Board of Management of the Wolverhampton and Staffordshire General Hospital, held on November 11, on the suggestion of the Medical Committee both the pathologists and the Medical Officer in Charge of the Venereal Department were elected to membership. To do this it was, however, necessary to alter the rules of the hospital, but fortunately in the early days of the war the General Board of Governors suspended the constitution, rules and regulations of the hospital, to enable the Board to cope with ever-recurring difficulties, and so left the conduct of the hospital entirely in the hands of the Board of Management. As a matter of fact, this particular election occurred just before the appearance in the *Times* on November 12 of the first paragraphs on the subject, and we trust that similar obviously necessary changes and modernisation are generally taking place without any promptings of the general Press.

### SINN FEIN AND MEDICAL APPOINTMENTS.

Even medical officerships in Ireland are matters of political controversy. Indeed it would seem that only genuine political passion can lend any attraction to the polls; for in its absence in all elections half the electors record no vote. In Ireland, may we not say, unfortunately, interest in elections is strong. The election of the medical officer of Omagh No. 1 dispensing district has excited so much interest that at the first poll the two opposing candidates received an equal number of votes. At the second polling eighty-three out of the eighty-four members of the Board of Guardians attended, and in consequence (we suppose) there was a majority of one for the medical officer favoured by the Sinn Feiners. How appalling it would be if the same energy was displayed in big constituencies so that the name of any abstaining voter was known!

### THE DUBLIN HOSPITAL CRISIS.

The inquiry which the Dublin Corporation has ordered into the closing of the Home of Industry hospitals, and into the withdrawal of the Treasury grant by which, however inadequately, they were largely maintained, will reveal the effects on the public health of this ill-considered action. Its effect upon other hospitals has been serious, and their condition is such that amalgamation is being discussed as a last resort for preserving them. The only satisfactory fact in the situation is that matters have reached a crisis, and since they could hardly be worse than at present it is legitimate to hope for a genuine effort to put them on their feet at last. Once again the voluntary principle has come to the rescue and has succeeded in partly reopening one of the three institutions.

### PRINCESS MARY'S HOSPITAL FOR CHILDREN.

The Metropolitan Asylums Board has received the Royal permission to re-christen East Cliff House, Cliftonville, Margate, Princess Mary's Hospital for Children. The Board has been busy of late years in re-christening its institutions by way of completing its many-sided activity. The title of the Board gives no idea to the public of the varied public work which it performs for many sections of the community, apart from the insane, and the day is probably not distant when the Board itself will be publicly recognised by a title more suitable than that which describes a work which it has long transcended.

### MOTHERS' PENSIONS.

A sessional meeting of the Royal Sanitary Institute is being held at 90 Buckingham Palace Road, S.W., on Friday, December 12, 1919, at 3 p.m., when a discussion will take place on "Mother's Pensions." The opening address will be delivered by H. Scurfield, M.D., the Medical Officer of Health for Sheffield.

### THE CLERGY AND ISOLATION HOSPITALS.

The Devizes Joint Isolation Hospital committee has been exercised in mind in the belief that most of the clergy are frightened to visit the place, in consequence of which the patients have to rely on the matron and her staff for "words of comfort." Since no record of the visits of the clergy has been kept, it is impossible to decide the matter privately. But it is fair to remember that clergy who are chaplains or have parishioners in isolation hospitals, have a double duty: to the infectious patient and to their flocks. Since, however, doctors are in the same position, and yet find that, with due precautions, they can come and go to infectious patients without becoming carriers of disease, it is reasonable to suppose that the clergy are in the like case, provided that the necessary precautions are explained to them, and that proper means are enforced upon them for carrying them out.

### HOSPITAL MONUMENTS AND THE PUBLIC.

Part of the season's programme of the London and County Rambling Society was a visit to Bethlem Hospital, where a lecture was given to members by the hospital chaplain, whose work is a recognised authority upon the history of the place. The plan apparently originated with the Society whose members attended the lecture, and afterwards made a tour of the building. But there is no reason why other hospitals of historical interests should not initiate similar visits, and thus make their traditions better known, attract new friends, and perhaps gather fresh subscriptions. We commend the suggestion at all events to every hospital which has, we will not say a history, but historical monuments, about which too little is generally known.

### A TRADESMAN'S GENEROSITY.

A tradesman lately supplied the Royal Albert Edward Infirmary, Wigan, with a large ward dresser, and complaints were made that the tiles were unsatisfactory and the dimensions wrong. Ultimately it was agreed that in view of the difficulty in obtaining satisfactory tiles nowadays, their replacement should remain in obeyance, but that the compartments should be altered to the sizes required. The give-and-take principle thus prevailed, but great was the surprise and pleasure of Mr. Lucas, the medical superintendent, when he was handed a cheque for £100, which the tradesman had decided, unknown to the former, to give to the infirmary if a conciliatory attitude were adopted. The cost of the dresser was £25, so it was not a case of refunding the profit! Thus are pounds won to a hospital even in minor disputes.

### PRESENTATION TO A HOSPITAL CHAIRMAN.

The Board of Nottingham General Hospital has presented to Mr. F. Acton an illuminated address congratulating him and his wife on their golden wedding, and in acknowledgment of Mr. Acton's valuable services as chairman during the five years of war. It is for more than twenty-six years that Mr. Acton has been a member of the hospital's board, and he was able to say in thanking the donors that the institution was now "a treat for anybody to see." The Duke of Portland made the presentation.

### THE RESCUE OF THE "KING EDWARD."

We had hoped that the days of danger to hospital ships were over, and are therefore sorry to read the Reuter telegram announcing the rescue of the British hospital ship *King Edward*, which was towed by a torpedo-boat into Christiansund last week. The vessel was on its way from Murmansk to London when it was caught in a storm and began to drift. By wireless she communicated with the Grip lightship, and when rescued was found to be almost without coal, and with every cabin full of water. So far as can be guessed from the report there was no loss of life or serious injury.

### A PROPOSED NOVEL AMALGAMATION.

There is great need for more hospital beds in West Fife, where the Dunfermline and West Fife Hospital is turning away patients, and funds for future extension are being raised. Meantime, however, the war has caused the casual to disappear, and the commodious Dunfermline Poorhouse, which was used as a military hospital, was lately evacuated in excellent condition by the Red Cross. To be precise, the latter occupied half the building, but the other half is practically a hospital since its inmates are all invalids. The decision rests with the parochial authorities; but surely the moment has come to form a scheme with the West Fife Hospital whereby the ex-poorhouse, now happily in hospital trim, can be used for patients in connection with the hospital, whose extension fund should be devoted to the equipment or endowment of the poorhouse. Difficulties always beset such schemes; but good-will can overcome them. Is this good-will common to the two institutions in West Fife?

### DEMOCRACY AND COMMON SENSE.

By a revision of its rules, which seems to have gained general assent, the East Suffolk and Ipswich Hospital has reduced the number of its board of management from one hundred to thirty. This step is so simple and has such obvious advantages that it says much for the ignorance of the community that it has not been possible to take it elsewhere. The confusion between "democracy" and mere numbers is still popular, so that in the north inflated committees are jealously safeguarded, and in natural consequence only by the promotion of an inner ring can anything be done. At Ipswich the body of governors elects the board, each member of which must offer himself or herself for re-election every three years. Within the board itself every section is represented. Thus the general body appoints the executive, which it can oust at the end of its three years' stewardship. In politics, as in household economy, servants are best controlled by the power of dismissal, not by constantly meddling in their work.

### CROYDON HOSPITAL AND SIGNS OF CHANGE

A testimonial is to be given to Mr. John Jones, the retiring secretary of Croydon General Hospital, who has held the position for forty-five years. Mr. E. W. Grimwade has started a fund, and the committee includes several prominent local names. Mr. Jones's retirement probably precedes the extension of the hospital, a step which he has helped it to take on important occasions in the past. Not only are more nurses' bedrooms required for the increased staff which is now necessary, but better rooms are wanted for Mr. Jones's successor. A really adequate extension would also, by relieving the waiting-list, do much to prevent the too early discharge of patients, an effect of which is to burden the local district nurses with more work than they are able to do. It is indeed no part of their duty to complete the treatment which the hospital undertakes to carry out. To such shifts and confusion has the shortage of beds reduced us that the voluntary system will have to pay dearly for this the chief weakness which has dogged it in the past.

### THE CONVERSION OF COTTAGE HOSPITALS.

A committee has been formed in conjunction with the committee of Colwyn Bay Cottage Hospital to establish a hospital for West Denbighshire at Colwyn Bay. For the scheme, which is expected to cost £25,000, the British Red Cross Society has promised £10,000 towards the new building. The Prince of Wales' Fund is also to be approached for a grant.

### A MATRON AS SECRETARY.

At the outbreak of the war, owing to the exposed position of the Yarrow Convalescent Home for Children at Broadstairs both from attacks from sea and from the air, the children were sent away and the Home placed at the disposal of the War Office authorities for a military hospital. The Home was finally vacated by the military in December 1917, and was reopened in August last for the reception of children. The special feature of the Yarrow Home is that it is for the children of gentle families of very limited means who now find themselves more or less in financial difficulties. Miss Chambers, who was formerly matron at the Home, has been appointed secretary at the London office, 6 Holborn Viaduct, E.C.

### THIS WEEK'S DRUG MARKET.

The persistent upward tendency of prices is one of the principal features of the drug market, and generally speaking can only be accounted for by the fact that the increase in the supplies has not been so marked as the increase in the demand. Several of the synthetic drugs, notably hexamine, resorcin and antifebrin have moved upward in price. The value of bromides is firmly maintained, and is likely to be so long as the imports continue to be so small. Somewhat unexpectedly prices of bismuth preparations have been advanced; this is not due to a rise in the price of the metal, but to the increase in other working costs; the advance in quotations is not substantial. Excessive prices are being paid for ergot of rye. Camphor, menthol and Japanese peppermint oil are again dearer. The scarcity of buchu leaves is unrelieved. Balsam tolu, podophyllum root, squill and senega root are dearer. Supplies of cocaine are scarce, and the price has an upward tendency. With the removal of the control on quinine, prices are likely to advance considerably.

